# Hospitality in the intensive care unit, welcoming the patient

**DOI:** 10.1186/2197-425X-3-S1-A723

**Published:** 2015-10-01

**Authors:** S Cabon, N Kentish-Barnes

**Affiliations:** Hospital Cochin, Paris, France; Saint Louis Hospital, Paris, France

## Introduction

Admission in the I.C.U. is a major but very challenging time to create hospitality. During those first few minutes, caregivers are absorbed by admission procedures and emergency treatments whereas patients experience a high level of anxiety and fear of death is often involved. I.C.U. patients have a high risk of developping PTSD and admission in I.C.U. can be a schock, but however, the notion of hospitality during the admission in intensive care is poorly studied.

## Objectives

To evaluate specificities of the welcoming moment in intensive care and assess how caregivers try and overcome difficulties.

## Methods

We conducted 16 semi-open interviews among ICU nurses from 3 different french MICUs. Each interview was conducted for 30-90 minutes using a semi-structured discussion guide to explore 5 main fields:

i) profile and experience of the nurses interviewed,

ii) anticipation of the patient admission,

iii) intensive care welcome and its specificities,

iv) place of the patient in the welcoming moment,

v) welcoming the family.

Analysis of the interviews involved the identification of obstacles to a satisfactory welcome in the I.C.U.

## Results

We interviewed 16 nurses (14 women, 2 men) with diverse ICU experience.

Analysis of interviews led to the identification of 5 primary obstacles:

i) an ubiquitous feeling of emergency,

ii) the threat of unexpected incidents,

iii) a very technical work,

iv) a heavy workload and

v) a high level of stress were expressed in all interviews.

Half of the caregivers interviewed added:

vi) their lack of experience and

vii) the crowd of caregivers present during some admissions.

We identified simultaneously strategies implemented by nurses to overcome those obstacles: trying and anticipate as much as possible, improve organisation, communicate with fellow caregivers, and mutual assistance.

## Conclusions

Caregivers are ambiguous: they like the « good » stress an emergency admission gives them, but they are conscious of the violence of an admission in the I.C.U. and they express their regrets about not being able to greet their patients in a more human way. The first moment of the welcome is a quick, standardized and technical instant during which caregivers have a lot of matters to concentrate on. Nevertheless, a second moment is often created later, which can become a long period if the patient is waking up from a coma and has cognitive issues.
